# Utilization of Digital Periodontal and Restorative Dentistry in Full Mouth Reconstruction: A Case Report

**DOI:** 10.7759/cureus.62469

**Published:** 2024-06-16

**Authors:** Badr Othman, Adham Abdulmajeed Tash Niyazi, Mohammed Alhusayni

**Affiliations:** 1 Periodontology Department, Faculty of Dentistry, King Abdulaziz University, Jeddah, SAU; 2 Prosthodontics Department, Batterjee Medical College, Jeddah, SAU; 3 Prosthodontics Department, King Fahad Hospital - Dental Speciality Center, Medina, SAU

**Keywords:** digital dentistry, periodontics, cbct, guided implant surgery, dental implants

## Abstract

CT-guided surgery has demonstrated superior accuracy over traditional methods in the dental literature. However, inherent errors such as discrepancies between jaw dimensions in scans and reality can challenge the fabrication of screw-retained provisional restorations. These discrepancies can impede final restoration fabrication. Traditional immediate loading of edentulous jaws using temporary cylinders in existing dentures often requires time-consuming abutment positioning and drilling. Many articles addressed these issues through minimally invasive implant placement with immediate loading, achieved through careful preoperative planning and exact prosthetic techniques. CT-guided surgery facilitates minimally invasive procedures and immediate restoration of edentulous jaws, resulting in reduced morbidity and quicker, more precise outcomes. This case report illustrates how digital dentistry enhances implant placement precision and reliability. It involves using a lock object system between the surgical guide and provisional restoration, streamlining the process. A 59-year-old male with significant periodontal issues and non-restorable teeth was treated with implant-supported fixed prostheses using digital planning and computer-fabricated surgical guides. The plan included immediate loading with mechanical and magnetic locks for optimal outcomes. The patient received complete fixed provisional restorations on both arches through minimally invasive procedures. Digital dentistry facilitated precise implant placement and restoration, improving function, esthetics, and patient satisfaction. Digital technologies streamlined the process, reducing time and enhancing predictability and reproducibility. In conclusion, integrating digital dentistry into implant treatment planning and execution offers enhanced accuracy, efficiency, and patient outcomes. By utilizing digital technologies and innovative methods, clinicians can attain consistent and reliable outcomes, thereby enhancing the quality of care for patients undergoing implant therapy.

## Introduction

CT-guided surgery has been extensively discussed in the dental literature [1]. It has superior accuracy compared to traditional methods [2]. Despite its benefits, inherent errors may persist, such as discrepancies between the size of the jaw in scans and its actual dimensions [3]. This can pose challenges in fabricating screw-retained provisional restorations based on surgical plans [4]. Discrepancies between the planned implant location and its actual placement curb the ability to fabricate the final restoration from the plan [5]. Past experiences have involved the immediate loading of edentulous jaws by incorporating temporary cylinders into existing dentures [6]. However, this process often requires time-consuming abutment positioning and drilling. To address these challenges, Baumgarten et al. demonstrated minimally invasive dental implant placement with immediate loading through meticulous presurgical planning, preparation, and precise prosthetic methods. CT-guided surgery enables minimally invasive implant procedures and immediate restoration of edentulous jaws, offering reduced morbidity and faster, more accurate outcomes for patients [7]. In this case, we illustrate how digital dentistry further enhances this process by enabling precise and reliable implant placement. This includes the incorporation of a lock object system between the surgical guide and provisional restoration, streamlining the process and ensuring optimal results.

## Case presentation

**Figure 1:**
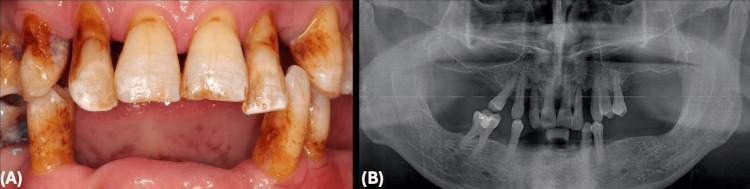
Initial Presentation (A) Clinical presentation at the initial visit. (B) Panoramic radiographic presentation at the initial visit

A 59-year-old male patient visited the clinic with complaints of tooth mobility and difficulty eating, indicating significant periodontal issues. Examination, including radiographs and cone beam CT scan (CBCT), confirmed the non-restorable condition of all teeth, prompting consideration for implant-supported fixed prostheses. The treatment plan involved digital planning and implant placement with computer-fabricated surgical guides, immediate loading, and utilizing a restoratively driven approach using a mechanical and magnetic lock for optimal, predictable, and reproducible surgical and restorative outcomes (Figure 1).

Digital planning phase

Digital Case Setup

**Figure 2:**
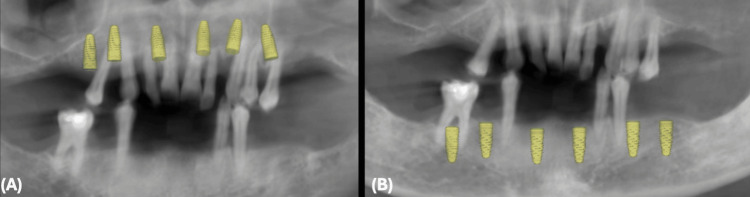
3D Planning of Dental Implants (A) Maxillary dental implant 3D planning. (B) Mandibular dental implant 3D planning

To effectively plan the patient's treatment, digital impressions of the maxilla and mandible along with bite registrations were obtained using the iTero intra-oral scanner (Align Technology, Inc., Tempe, Arizona). These files were then digitally forwarded to the Doxel Dental Laboratory in Jeddah, Kingdom of Saudi Arabia (KSA), where digital wax-ups were designed and fabricated. This allowed for precise determination of tooth position, smile esthetics, and optimal implant placement. Subsequently, a CBCT scan was performed on both jaws, and the digital imaging and communications in medicine (DICOM) files were imported into implant-planning software (coDiagnostiX; Dental Wings GmbH, Chemnitz, Germany) (Figure 2).

**Figure 3:**
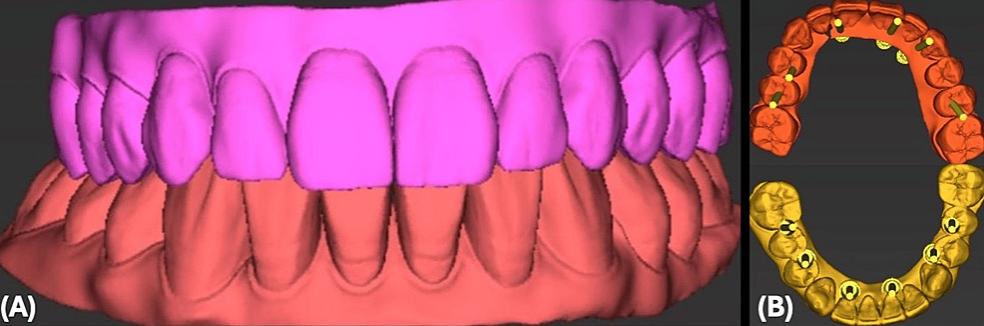
Restoratively Driven Implant Planning (A) Digital diagnostic wax-up for the maxilla and mandible. (B) Access holes for dental implants in maxillary and mandibular diagnostic wax-ups

The digital wax-ups were merged with the DICOM files, and virtual implants and fixation screws were strategically placed to achieve a restoratively driven outcome, considering bone availability (Figure 3).

Surgical guides were then designed, and the digital wax-ups were digitally hollowed for the planned implant-supported provisional restorations.

Mechanical and Magnetic Objects Designing

**Figure 4:**
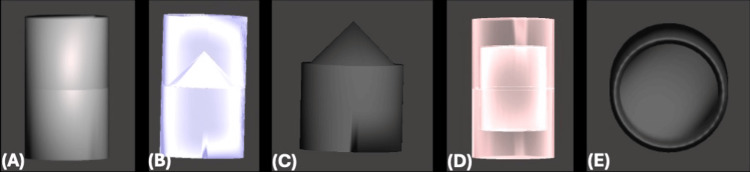
Objects Designed on Meshmixer (A) Upper and lower parts locked together as one solid object. (B) A transparent view showing the mechanical lock from the lower male part engaging in the upper female part. (C) The design of the mechanical lock object. (D) A transparent view showing the magnetic holding force lock as the lower and upper parts are aligned. (E) The design of the magnetic holding force lock object.

All files were then imported to Autodesk Meshmixer (version 3.5) where mechanical and magnet lock objects were placed to ensure reproducible interchangeable positioning between surgical guides (Figure 4).

Maxillary Surgical Guide Positioning and Fixation Designing

**Figure 5:**
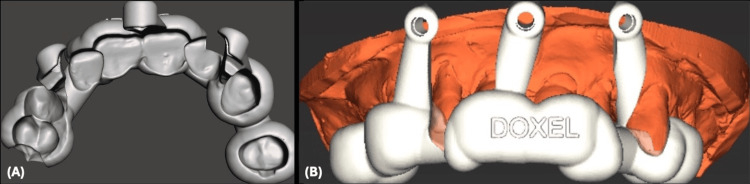
Maxillary Surgical Guide Positioning and Fixation Designing (A) Virtual view of maxillary starter surgical guide. (B) Virtual view of the maxillary starter surgical guide designed to be fitted on the maxillary standard triangle language (STL) model.

The patient's teeth were utilized as an initial reference point, and a starter surgical guide with its fixation sleeves was designed to be fitted over the maxillary standard triangle language (STL) model (Figure 5).

**Figure 6:**
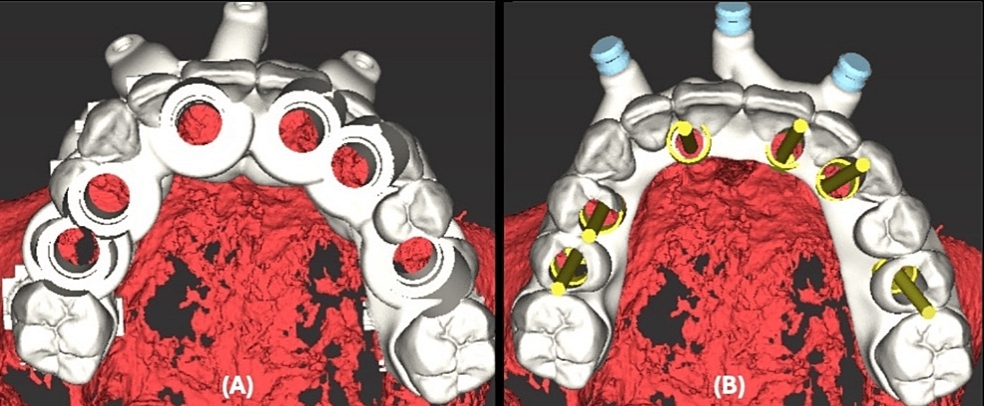
Maxillary Implant Surgical Guide and Implant-Supported Provisional Restoration Design (A) Maxillary implant surgical guide designed with fixation holes. (B) Maxillary implant-supported provisional restoration hollowed digitally and designed with fixation holes.

After the virtual extraction of the remaining hopeless teeth, the implant surgical guide and the implant-supported provisional restoration were also designed with fixation holes to be secured later on with the same fixation pin (Figure 6).

**Figure 7:**
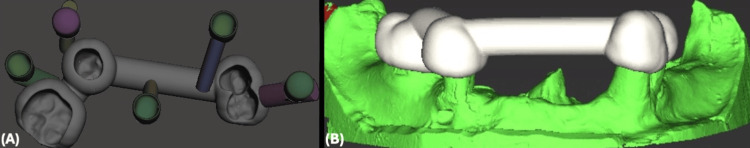
Mandibular Surgical Guide Positioning and Fixation Designing (A) Virtual view of the mandibular starter surgical guide. (B) Virtual view of the mandibular starter surgical guide designed to be fitted on the mandibular STL model.

Mandibular Surgical Guide Positioning and Fixation Designing:

Similar to the maxillary procedure, the patient's teeth were utilized as a reference point, and a starter surgical guide was designed to be fitted over the mandibular STL model (Figure 7).

**Figure 8:**
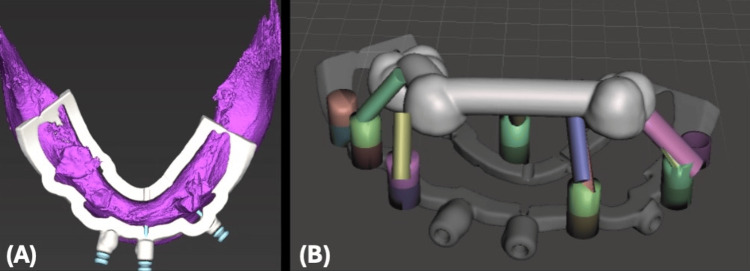
Virtual Design of the Starter Surgical Guide and Stackable Fixation Base in the Mandible (A) Virtual design of the stackable fixation base in the mandible. (B) Virtual stackable fixation base locked with a mandibular starter surgical guide.

A stackable surgical guide base was designed with multiple lock objects mechanically through male and female lock objects and magnetically through magnets for strong holding force. A stackable surgical guide base was aligned with a mandibular starter surgical guide (Figure 8).

**Figure 9:**
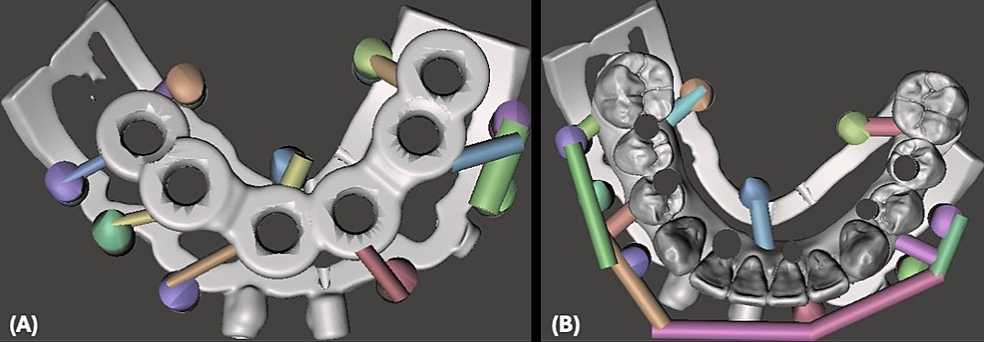
Mandibular Implant Surgical Guide and Implant-Supported Provisional Restoration Design (A) Mandibular implant surgical guide designed to be aligned with the stackable guide base. (B) Mandibular implant-supported provisional restoration hollowed digitally and designed to be aligned with the stackable guide base.

After the virtual extraction of the remaining hopeless teeth, the implant surgical guide and implant-supported provisional restoration were also designed to be aligned and fitted over the stackable surgical guide base and securely locked by the mechanical lock and magnets holding force objects (Figure 9).

All the files were then 3D-printed and sleeves and magnets were securely inserted.

Surgical phase

Treatment was carried out in two phases in one session, with the maxilla addressed during the first phase and the mandible during the second. Each phase involved anesthesia using 2% xylocaine with 1:100,000 epinephrine.

Maxillary Surgical Guide Positioning and Fixation

**Figure 10:**
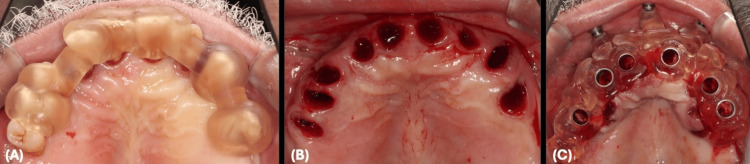
Maxillary Surgical Guide Positioning Clinically (A) Starter surgical guide fitted over the maxillary teeth to drill fixation pins. (B) The starter surgical guide was removed and maxillary teeth were extracted. (C) The implant surgical guide is fixated with fixation pins.

The patient's teeth were utilized as an initial reference point, and a starter surgical guide with its fixation sleeves was placed and fitted over the teeth perfectly. A fixation drill was used to drill through the fixation sleeves directly to the maxillary bone. The stater surgical guide was then removed and followed by teeth extraction and subsequent fixation of the implant surgical guide in the same position using 13-mm-long fixation pins (Figure 10).

Mandibular Surgical Guide Positioning and Fixation

**Figure 11:**
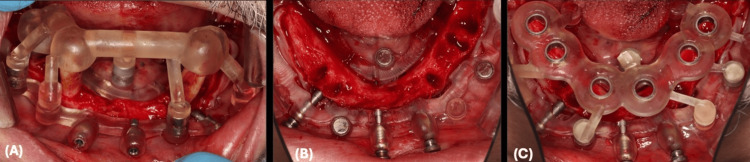
Mandibular Surgical Guides Positioning Clinically (A) Starter surgical guide fabricated and fitted over mandibular teeth to guide the position of the stackable fixation base through mechanical and magnets lock objects. (B) The stackable fixation base is fixated, the starter guide was removed, and mandibular teeth were extracted. (C) Mandibular implant surgical guide locked over the stackable fixation base.

Similar to the maxillary procedure, the patient's teeth were utilized as a reference point, a starter surgical guide was fitted over the teeth, and then a stackable surgical guide base was positioned through multiple lock objects mechanically through male and female lock objects and magnetically through magnets strong holding force. The stackable surgical guide base was secured to the bone after drilling fixation holes and using 13-mm-long pins. The starter surgical guide was removed and followed by teeth extraction. The implant surgical guide was then fitted perfectly over the stackable surgical guide base and securely locked by the mechanical lock and magnets holding force objects (Figure 11).

Implant Placement

**Figure 12:**
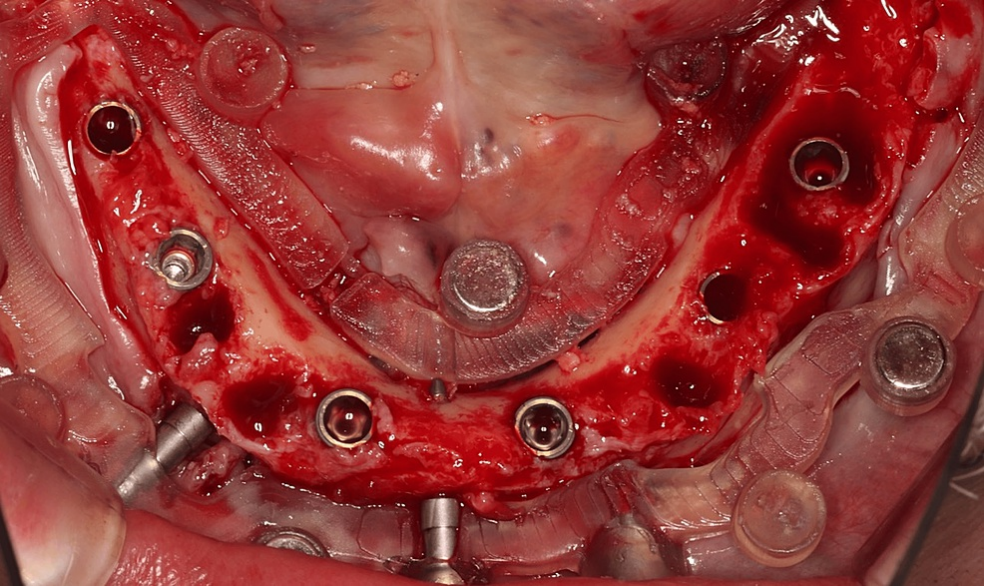
Implant Placement Clinical presentation of the dental implant placement in the mandible.

Guided drills were employed to prepare the osteotomies, and dental implants (Nobel BioCare Tapered Conical Connection, Kloten, Switzerland) were inserted through the sleeves of the surgical guide using guided implant mounts with high stability (35 Ncm) (Figure 12).

Immediate Loading

**Figure 13:**
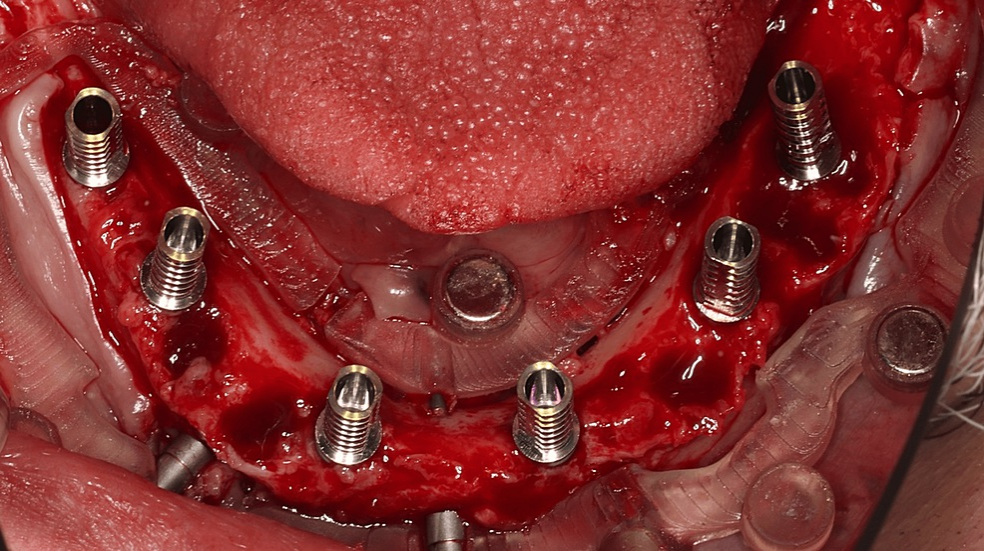
Temporary Abutments Placed the Over Dental Implants Clinical presentation of the temporary abutment placement in the mandible.

Temporary abutments were secured to the implants with gold prosthetic screws, and a rubber dam was placed to isolate the surgical site (Figure 13).

**Figure 14:**
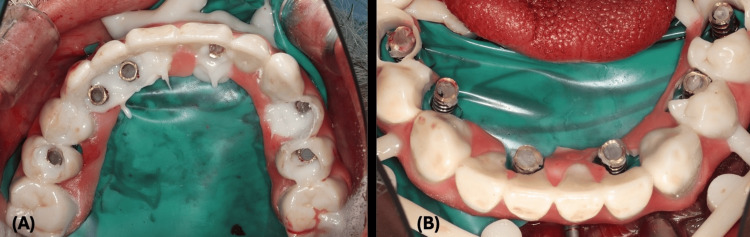
Implant-Supported Provisional Pickup (A) Maxillary implant-supported provisional restoration picked up with temporary abutments. (B) Mandibular implant-supported provisional restoration picked up with temporary abutments.

The provisional restoration, prefabricated with holes for easy retrieval, was affixed to the temporary abutments with composite core build-up material (Figure 14).

**Figure 15:**
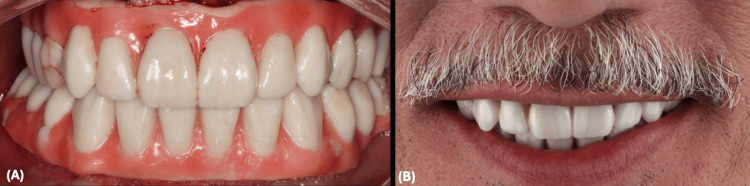
Immediate Loading (A) Implant-supported provisional restorations' immediate loading. (B) Patient's smile on surgery day.

After adjusting the occlusion, the prosthesis was unscrewed, finished in the laboratory, and loaded to the patient mouth. The patient was placed on soft diet during the three months of healing phase and prescribed antimicrobial rinse, antibiotic, and analgesic medications (Figure 15).

Final restoration phase

**Figure 16:**
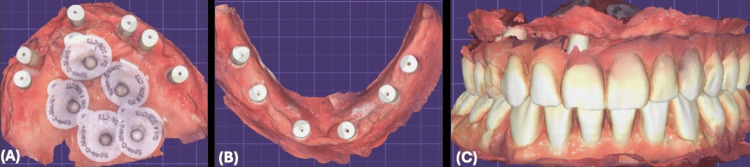
Digital Final Impressions (A) Maxillary digital impression with intraoral scan bodies. (B) Mandibular digital impression with intraoral scan bodies. (C) Digital impression of implant-supported provisional restoration.

After three months, the provisional restorations of maxilla and mandible and bite registrations were scanned intra-orally using the iTero intra-oral scanner as a diagnostic record to be used during the design of the final restoration. Intra-oral scan bodies were placed over the implants, scan markers were placed over the soft tissue, and then an implant-level intraoral scan was done for the maxilla and mandible to capture implant positions (Figure 16).

**Figure 17:**
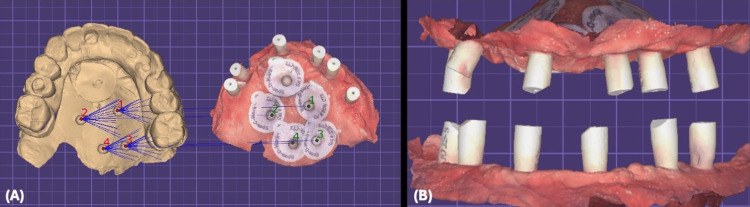
Digital Work Flow (A):- Digital merge of scan markers (B):- Digital mounting and bite registration

All files were sent to the dental laboratory to be merged, digitally mounted, and designed for the final restoration (Figure 17).

**Figure 18:**
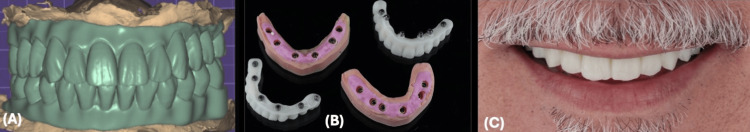
Prototype Try-In (A) Digital wax-up of the final restoration. (B) 3D printing of master casts with digital analogs and prototypes. (C) Prototype try-in.

3D printing of the master cast from the digital impression digital was done with soft tissue coverage, and digital analogs were placed. A prototype was then milled over non-engaging titanium abutments to be verified and tested for the fit, occlusion, esthetics, and phonetics (Figure 18).

**Figure 19:**
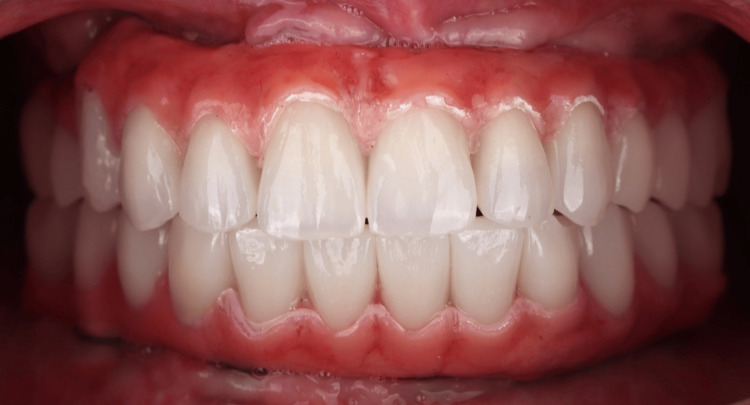
Implant-Supported Restoration Insertion

The final implant screw retained final restoration was then milled and delivered to the patient mouth (Figure 19).

Results and clinical outcomes

**Figure 20:**
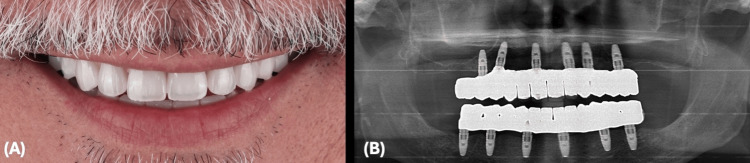
Clinical Outcome (A) Patient's smile at delivery. (B) Final panoramic radiograph.

The patient underwent successful placement of complete fixed provisional restorations on both arches, secured by implants inserted through minimally invasive surgical procedures. Utilization of digital dentistry allowed for precise implant placement, starting from utilizing the patient's teeth as the initial reference point and incorporating a lock object system within the stackable base guide, implant surgical guide, and provisional restoration. Furthermore, digital impression technology enhanced the patient experience by providing greater comfort and convenience while ensuring more precise treatment outcomes (Figure 20).

## Discussion

The present study highlights the utilization of digital dentistry in enhancing the predictability and efficiency of dental implant placement and restoration procedures in a 59-year-old male patient with significant periodontal issues. This comprehensive approach involved meticulous planning and execution, incorporating various digital technologies and innovative techniques to achieve optimal outcomes.

In this case report, it is essential to discuss patient selection criteria for the treatment approach undertaken. Absolute contraindications for this treatment include osteopathies and disorders of bone metabolism, renal insufficiency and uremia, liver disease, hyperthyroidism, hypopituitarism, connective tissue diseases, specific autoimmune diseases such as lupus, leukopoietic and erythropoietic diseases, and high-dosage radiotherapy. Relative somatic conditions that must be considered are osteoporosis, aggressive rheumatoid arthritis, treated endocrine disorders, anticoagulant treatment, drug and alcohol abuse, and low-dosage radiotherapy. Temporary contraindications also need to be addressed, including transient infections, systematic therapies affecting wound healing, anticoagulant or immunosuppressive therapy, corticosteroids, and chemotherapy.

Additionally, the patient's pre-existing periodontal conditions should be considered, emphasizing the importance of rigorous peri-implant maintenance to ensure optimal outcomes. In this particular case, although the patient had periodontitis, they still had a sufficient maxillary and mandibular bone for implant placement, eliminating the need for grafting or sinus lift procedures. Highlighting that this treatment method is suitable only for patients with adequate bone structure, even in the presence of periodontitis will help identify appropriate candidates more effectively and we can ensure that the treatment is applied to patients who are most likely to benefit while minimizing potential risks and complications. This comprehensive approach will contribute significantly to the success and reliability of the treatment outcomes in similar future cases.

Digital treatment planning

Digital impressions of the maxilla and mandible, along with bite registrations, were obtained using the iTero intra-oral scanner. This initial step allowed for precise determination of tooth position, smile esthetics, and optimal implant placement. The integration of digital wax-ups and CBCT scans further facilitated treatment planning, enabling the creation of a restoratively driven outcome considering bone availability. The use of implant-planning software allowed for the virtual placement of implants and fixation screws, ensuring accurate positioning for optimal surgical and restorative outcomes. Digital impressions capturing the entire arch represent a viable and dependable substitute for traditional impression methods [8]. Among young orthodontic patients, digital impressions emerged as the most preferred and comfortable impression method when contrasted with conventional techniques [9]. The digital impression taken in this case shortened the overall process by capturing all files digitally and allowing them to be utilized and merged through the implant planning software. This process eliminated the need for conventional impression pouring the desktop scanning in the dental laboratory minimizing the time, cost, and pouring distortion.

Surgical phase

The surgical phase involved the utilization of computer-fabricated surgical guides, which were custom-designed based on the digital treatment plan. These guides provided precise guidance for implant placement, minimizing surgical trauma and reducing the risk of errors. In the maxillary and mandibular surgeries, the utilization of a lock object system between the surgical guide and provisional restoration ensured reproducible and interchangeable positioning, enhancing predictability and reliability. In the case of total arch dental implant placement with immediate loading, guided dental implant surgery is crucial. Clinicians typically take the surgical guide support from soft tissue or bone during dental implant placement. However, extraction of hopeless dentition and the changes in anatomical structures complicate the accurate positioning of the surgical guide. Additionally, delivering immediate provisional restoration is crucial because of the surgical and restorative phases of workflow discontinuity [10].

The process of designing and manufacturing a stackable implant surgical guide, incorporating magnetic retention, within a digital workflow is outlined. This methodology aims to enhance both the stability of the surgical guide stack and the precision of implant positioning [11]. The deteriorating dentition in partially edentulous patients can be utilized as a starting reference for surgical guides in full-mouth dental implant placement. Using a stackable guide fabricated from a digital workflow improves the predictability of bone reduction and immediate dental implant placement with immediate loading. A practical method for designing and creating a metal framework with occlusal rests enhances the use of a tooth-supported surgical guide for full-arch immediate implant placement in patients with failing dentition, resulting in increased stability and improved precision [12]. The survival rates for dental implants placed and restored by students are similar to those achieved by experienced dentists, indicating the high precision of the procedures [13].

The technique errors should also be respected. A review paper found a deviation of 1 mm (apex), 1.5 mm (entry point), and an angular deviation of five degrees [14]. The maximum recognized deviation should be used to establish the safety zone during implant placement, typically ranging from 1 to 1.5 mm. Errors can occur at various stages, including patient positioning during CBCT exposure, impression taking or pouring, digital file merging, printing or milling, clinical fixation of the surgical guide, and drilling [1]. In our case, we used the patient STL to fabricate the starter guide as the most reliable fit, followed by stackable guides with mechanical and magnetic locks with holding force and fixation pins. This technique made the procedure smooth and predictable. 

Immediate loading protocol

Immediate loading of implants was performed, further streamlining the treatment process and providing immediate functional and esthetic benefits to the patient. Temporary abutments were secured to the implants, and prefabricated provisional restorations were immediately placed, allowing the patient to benefit from restored dentition during the healing phase. This immediate loading protocol, combined with meticulous surgical and prosthetic techniques, contributed to favorable clinical outcomes and enhanced patient satisfaction. Utilizing a reference template to transfer the digital plan to the surgical field has improved the accuracy and seamless integration of the surgical and prosthetic phases throughout the entire workflow [15]. The remarkable accuracy of implant placement using a surgical template from preoperative virtual planning guarantees a relatively short treatment time and a smooth, rapid recovery with minimal discomfort. Additionally, immediate prosthodontic rehabilitation benefits both the patient and the dental team [16]. In a clinical series involving dental implants and immediate loading for the management of edentulous patients, the results demonstrated a 100% survival rate for both implants and prosthetics. The study indicated that the protocol can be used with a high level of anticipated success [17].

Final restoration phase

After the healing phase, the provisional restorations were replaced with final screw-retained restorations. Digital impression technology was once again utilized to capture intraoral scans for the design and fabrication of the final restorations. The use of 3D printing and digital analogs allowed for precise fit and accurate replication of the digital treatment plan. The final restorations were meticulously tested for fit, occlusion, esthetics, and phonetics before being delivered to the patient, ensuring optimal function and esthetics. Based on primarily in vitro studies, digital scans demonstrate similar 3D accuracy to conventional implant impressions. Nevertheless, it is advisable to conduct clinical trials to assess the clinical accuracy of digital scans and interim prostheses fabricated digitally before advocating for the routine clinical adoption of digital implant scans [18]. As intraoral scanners continue to evolve, further improvements in accuracy are anticipated. Additionally, ongoing verification of their accuracy is essential [19]. The results of another review indicated that conventional impressions are more precise than digital impressions. However, further research studies need to be done to evaluate the accuracy of digital impressions across a broader range of clinical scenarios [20]. In other in-vitro studies, digital impressions showed superior marginal and internal fit for fixed prostheses compared to conventional methods [21]. In our case, we went through the prototype verification to ensure precision. Digital impressions appear to be a predictable way for fabricating full-arch dental implant frameworks, ensuring a satisfactory passive fit even when tilted implants are used [22].

Clinical outcomes

The patient underwent successful placement of complete fixed provisional restorations on both arches, secured by implants inserted through minimally invasive surgical procedures. The utilization of digital dentistry allowed for precise implant placement and restoration, resulting in improved function, esthetics, and patient satisfaction. Furthermore, the incorporation of digital technologies streamlined the treatment process, reducing treatment time and enhancing treatment predictability and reproducibility.

## Conclusions

In conclusion, the integration of digital dentistry into implant treatment planning and execution offers numerous advantages, including enhanced accuracy, efficiency, and patient outcomes. By leveraging digital technologies and innovative techniques, clinicians can achieve predictable and reproducible results, ultimately improving the quality of care for patients undergoing implant therapy.
